# Specialists’ Perceptions of Workforce Retention Strategies in the Malaysian Ministry of Health and Their Association With Job Satisfaction and Turnover Intention: Protocol for a REDCap-Based National Cross-Sectional Survey

**DOI:** 10.2196/83377

**Published:** 2026-04-01

**Authors:** Siti Zubaidah Ahmad Subki, Ili Abdullah Sharin, Nor Haniza Zakaria, Nursyahda Zakaria, Pangie Bakit, Izzuan Khirman Adnan, Norehan Jinah, Kun Yun Lee

**Affiliations:** 1 Institute for Health Management, National Institutes of Health, Ministry of Health Malaysia, Block B1, NIH Complex, No. 1 Jalan Setia Murni U13/52, Section U13, Setia Alam, Shah Alam, 40170, Malaysia, 60 333628314

**Keywords:** physician, job satisfaction, turnover intention, retention strategies, sustainability, REDCap

## Abstract

**Background:**

Retention of specialists is critical for sustaining health system performance. In Malaysia, the Ministry of Health (MOH) has implemented multiple workforce retention strategies (WRSs) to enhance job satisfaction and reduce turnover among specialists. However, evidence on specialists’ awareness, participation, and perceived effectiveness of these strategies remains limited. To address this gap, we plan to administer a standardized survey among specialists in MOH health care facilities to guide evidence-based strategic planning toward specialist retention.

**Objective:**

This protocol describes a national-level online survey to assess MOH specialists’ perceptions of WRS and examine their associations with job satisfaction and turnover intention, using REDCap (Research Electronic Data Capture) to ensure real-time data collection within a robust and secure digital platform.

**Methods:**

A cross-sectional mixed methods study will be conducted among 1325 MOH specialists selected through systematic random sampling from the Human Resource Management Information System. Data collection will be implemented using a REDCap-based workflow to support secure and efficient survey administration. REDCap functionalities will be used, including automated survey invitations with nontransferable links to prevent duplicate responses, branching logic to tailor item relevance, auto-reminder scheduling, and real-time data validation to minimize entry errors. The self-administered questionnaire comprises measures of specialists’ awareness, participation, and perceived effectiveness of 12 MOH-implemented WRSs, job satisfaction assessed using the Job Satisfaction Survey, and turnover intention measured with the Turnover Intention Scale-6, as well as 2 optional open-ended questions for qualitative input to strengthen the quantitative results. A pilot study will be conducted to assess instrument reliability and REDCap platform usability. Quantitative data will be exported from REDCap for descriptive and inferential analyses, while qualitative responses will undergo thematic analysis using NVivo and be integrated with quantitative findings during interpretation.

**Results:**

This study will generate vital evidence of MOH specialists’ engagement with WRSs and how perceptions of these strategies related to their job satisfaction and turnover intention. Participant recruitment and data collection have been completed, and the study is in the data analysis phase. It is expected that the data analysis will be completed in March 2026 and that the results will be published in June 2026.

**Conclusions:**

This protocol establishes a REDCap-based survey for conducting large-scale research focused on MOH specialists. Findings are expected to guide data-driven improvements to WRS implementation within the MOH Malaysia and may serve as a useful methodological model for similar research in public sector workforce studies.

## Introduction

### Background

Retention strategies are essential for building a sustainable and efficient workforce, particularly in the health care sector, where skilled professionals play a pivotal role in delivering high-quality services [1]. These strategies aim to address ongoing workforce shortages and promote long-term stability by fostering a motivated and committed workforce. As service demands grow and global shortages of health care professionals intensify, effective retention strategies have become increasingly important [1]. Specialists, in particular, are often the focus of retention strategies due to their roles in complex clinical decision-making, procedural care, and mentorship of junior staff [2]. Retaining these highly trained professionals supports continuity of care and reduces the substantial costs associated with recruitment and training [3].

Globally, retention strategies vary depending on a country’s economic capacity, health care priorities, and system infrastructure. High-income countries such as the United Kingdom often adopt large-scale approaches like international recruitment, supported by mentorship programs and relocation incentives. However, these strategies frequently face limitations such as high implementation costs, difficulties with professional integration, and limited long-term retention [4,5]. Middle-income countries, including Brazil and Thailand, typically implement targeted strategies such as bonded scholarships, rural service allowances, and performance-based incentives to address workforce shortages in underserved areas [6,7], though these often struggle with administrative complexity and resource constraints. In contrast, low-income countries tend to rely on donor-funded programs and compulsory service schemes, which are frequently undermined by inadequate infrastructure and limited career progression opportunities [8]. These global experiences highlight the importance of context-sensitive, multifaceted strategies that reflect the unique dynamics of each health system [9,10].

In this context, Malaysia has introduced various workforce retention strategies (WRSs) since the early 1990s to improve the retention of specialists in the Ministry of Health (MOH) (see Multimedia Appendix 1; Human Resource Division, Ministry of Health Malaysia, unpublished data, 2023). These strategies encompass financial incentives (eg, on-call allowances, specialist incentive allowance, and Full Paying Patient Scheme), structured career progression pathways (eg, time-based promotion and introduction of Grade 56), flexible working arrangements (eg, flexible working hours), and professional development opportunities (eg, subspecialty training and short-term training abroad). In addition, geographically based allowances are used to support the deployment of specialists to underserved regions, such as Sabah and Sarawak. These initiatives are consistent with international best practices, which emphasize a combination of remuneration, professional support systems, and career advancement opportunities tailored to local workforce dynamics [11].

Although these strategies represent a multifaceted approach to workforce retention, patterns in specialist resignation trends have varied over time. Administrative data from the MOH Human Resource Division showed that overall resignation rates among specialists fluctuated between 2.1% and 5.8% from 2015 to 2023 (Human Resource Division, Ministry of Health Malaysia, unpublished data, 2023). Between 2015 and 2017, observed reductions coincided with the implementation of several major WRSs, including the Full Paying Patient Scheme, the introduction of Grade 56, and flexible working hours (Human Resource Division, Ministry of Health Malaysia, unpublished data, 2023). During this period, overall resignation rates declined from 3.9% to 3.4%, with a more substantial drop observed among surgical specialists, whose rates fell from 6.7% to 4.6% (Human Resource Division, Ministry of Health Malaysia, unpublished data, 2023). While these temporal patterns coincide with the introduction of selected WRSs, the descriptive nature of these data does not permit the attribution of changes in resignation trends to specific strategies. Rising resignation rates in subsequent years underscore the need to examine how contextual factors and implementation conditions of WRSs are associated with specialists’ perceptions of the effectiveness and sustainability of these strategies.

To date, there has been a limited systemwide examination of specialists’ uptake, engagement, and perceptions of WRSs in MOH, Malaysia, since their inception. Existing studies tend to be narrow in scope, often focusing on individual strategies in isolation, which limit their generalizability and practical relevance for broader policy development [12,13]. A more comprehensive assessment of how specialists engage and how they perceive these strategies in relation to job satisfaction and turnover intention is therefore warranted [14,15]. To guide this assessment, theoretical frameworks offer valuable insights for understanding the relationships between retention strategies and workforce-related outcomes. One of the most widely used is Herzberg’s Two-Factor Theory, which distinguishes between hygiene factors (eg, salary, job security, and working conditions), which prevent dissatisfaction, and motivators (eg, recognition, professional growth, and autonomy), which enhance job satisfaction and promote retention [16,17]. This theory underpins models such as Australia’s Whole-of-Person Retention Improvement Framework and is supported by systematic reviews on health care workforce stability [14,18]. Applying this framework to Malaysia’s public health context may help clarify which components of WRSs are most effective in supporting long-term workforce sustainability.

To address current evidence gaps, a systematic and scalable data collection approach is required. REDCap (Research Electronic Data Capture) is a secure, browser-based platform with customizable survey logic, encryption, audit trails, and real-time validation features that are ideal for online data collection. It has been used in over 140 countries, with a proven track record in supporting large-scale research [19,20]. Despite its widespread use in clinical and epidemiological studies, REDCap remains underutilized for workforce policy-related research, especially in middle-income countries [21]. Its application in this study will enable standardized, confidential, and geographically distributed data collection across Malaysia’s public health system, critical for assessing specialist engagement with WRSs, as well as offering a methodological framework for similar research.

### Objectives

This protocol will describe a national-level survey using REDCap to systematically examine Malaysian MOH specialists’ awareness, participation, and perceptions of 12 MOH-implemented WRSs. It will outline the methodological approach for examining associations between these engagement dimensions, job satisfaction, and turnover intention. It is intended to support standardized data collection and generate reproducible evidence to inform policy and practice for strengthening specialist retention in Malaysia’s public health care system.

## Methods

### Study Design and Setting

This study will adopt a nationwide, cross-sectional design using a closed online survey administered through the REDCap platform, hosted by the Sector of Biostatistics and Data Repository, National Institutes of Health Malaysia. The study design was developed based on REDCap version 14.0.21. Data collection was conducted using the institutionally supported current long-term support version of REDCap. All study procedures will be centrally administered under a single governance structure, with participants drawn from multiple MOH facilities in the country. To ensure methodological rigor and reporting transparency, the study will be conducted in accordance with the CHERRIES (Checklist for Reporting Results of Internet E-Surveys (Checklist 1) and guided by the STROBE (Strengthening the Reporting of Observational Studies in Epidemiology) checklist.

### Study Timeline

The study will be conducted over a 24-month period. All stages, from initial recruitment to data analysis and reporting, will be carried out systematically within this timeframe. The timeline will be structured to allow sufficient time for comprehensive data collection, analysis, and interpretation. A detailed breakdown of the study phases is provided in Table 1.

**Table 1.** Study timeline.

Timeline	Activity
Months 1‐3	Recruitment of study team members Planning of the full study
Months 4‐6	Pilot study commencement
Months 7‐10	Refinement of the questionnaire based on pilot test findings Stakeholders’ feedback
Months 11‐13	Nationwide data collection
Months 14‐20	Data analysis and synthesis
Months 21‐24	Result dissemination Preparation of the final report

### Study Population

The study population will comprise all 8953 MOH specialists actively listed in the Human Resource Management Information System 2.0 (HRMIS 2.0) as of December 2023. This time point will be selected to ensure the use of the most current and complete national dataset available prior to sampling and survey distribution. HRMIS 2.0 is the official centralized platform for managing civil service personnel records in Malaysia and is widely used for workforce planning, administrative monitoring, and strategic human resource deployment [22,23]. The system provides standardized information on employment status, job designation, and career trajectory and is accessible via a secure web interface [24]. Leveraging HRMIS 2.0 as the sampling frame will enable comprehensive, nationally representative coverage of the MOH specialist workforce, minimize selection bias, and enhance participant identification accuracy. This approach aligns with best practices in health workforce research and ensures that the sample reflects the diversity of specialist roles and geographic distribution within Malaysia’s public health care system. Inclusion and exclusion criteria will subsequently be applied to refine the study sample in accordance with the research objectives.

### Inclusion Criteria

The study will include all MOH specialists with full registration who are actively serving in MOH facilities, including hospitals, health clinics, district health offices, state health departments, specialized institutes, and the MOH headquarters. Inclusion across a wide range of health care settings is intended to ensure comprehensive coverage of specialists.

### Exclusion Criteria

Dental specialists, specialists who have not yet obtained full registration (eg, those under gazettement), contract-based specialists, and those on temporary leave will be excluded. Dental specialists are excluded due to differences in practice context, while the remaining criteria ensure the inclusion of individuals eligible for and directly covered by MOH WRSs.

### Sample Size Calculation and Sampling Method

The sample size will be calculated based on a 95% CI (*z*=1.96), a 5% margin of error (*d*=0.05), and an anticipated turnover intention rate of 46.8% among MOH specialists, as reported in prior studies [12]. This results in a minimum required sample size of 382 participants. After adjusting for an anticipated 40% nonresponse rate, which is commonly observed in web-based surveys, the sample size will be increased to 637 participants to ensure an optimum-achieved sample size [25]. The sample size was inflated by 40% to ensure that the final number of participants who complete the study meets the minimum required sample size for the planned statistical analyses and power considerations.

Considering 2 main groups of specialists (surgical and nonsurgical), the adjusted sample size will be doubled, resulting in a total of 1274 participants (637×2). Based on national workforce distribution data from the Human Resource Division and Medical Development Division, MOH Malaysia, the proportions of nonsurgical and surgical specialists are 74% and 26%, respectively. Applying these proportions to the adjusted sample yields an estimated requirement of 943 nonsurgical specialists (1274×0.74) and 331 surgical specialists (1274×0.26). However, the estimated sample size for the surgical subgroup (n=331) falls below the minimum sample size requirement of 382 derived from the initial prevalence-based calculation; therefore, it is adjusted to 382. Consequently, the final target sample size for this study is 1325 participants.

Participant recruitment will be conducted using the list of specialists in active service obtained from the Human Resource Department, MOH, which is provided in alphabetical order. The HRMIS list will be randomly shuffled using Excel=RAND() before the systematic sampling interval is applied. Following this, a random starting point will be generated for each stratum, followed by a fixed sampling interval. Participants will be selected using systematic random sampling within each specialty stratum (surgical and nonsurgical). To achieve the required samples of 943 nonsurgical and 382 surgical specialists, sampling intervals will be set at 1 in every 7 nonsurgical specialists and 1 in every 6 surgical specialists, reflecting the relative size of each stratum.

### Study Tool

#### Questionnaire

A structured questionnaire will be developed by adapting items from previously validated instruments, including items from Belbin et al [15] to assess retention strategies and the Job Satisfaction Survey (JSS) [26,27] and the Turnover Intention Scale-6 (TIS-6) [28] to measure job satisfaction and turnover intention, respectively. These adaptations aim to ensure conceptual alignment with the study objectives, support content relevance for the target population, and contextualize the instrument for use among MOH specialists in Malaysia. To enhance clarity, contextual appropriateness, and usability, the draft questionnaire will undergo face and content validation by key stakeholders and subject matter experts. Feedback from this process will inform refinements to item wording, logical sequencing, and response formatting. Internal consistency of multi-item domains will be assessed during the pilot phase to guide potential scale refinement. The finalized questionnaire will comprise 5 sections, as summarized in Table 2, and will be administered using the REDCap platform to ensure secure and efficient online distribution. It will include both closed-ended items (eg, Likert-scale and multiple-choice formats) and optional open-text fields to capture contextual insights, thereby supporting a mixed methods analysis approach.

**Table 2.** Questionnaire items and measures.

Survey and question number	Type	Responses
Section A: awareness, participation, and perceived effectiveness of 12 WRSs^a^ (13 items)		
a. Awareness: Q 1(a) to 12(a)	Dichotomous scale	Yes or no
b. Participation: Q 1(b) to 12(b)	Dichotomous scale	Yes or no
c. Effectiveness Q 1(c) to 12(c)	Interval scale	1=effective; 2=slightly effective; 3=not effective; 4=don’t know
Q 13	Open-ended questions	Free text (“Any suggestion for other retention strategies that might be beneficial?”)
Section B: individual and organizational WRS barriers (3 items)		
Q 1 to 2	Interval scale (5-point Likert scale)	1=strongly disagree; 2=disagree; 3=neutral; 4=agree; 5=strongly agree
Q 3	Open-ended questions	Free text (“Any other barriers that could prevent you from participating in any retention strategies?”)
Section C: Job Satisfaction Survey (JSS) (36 items classified into 9 domains, 4 items per domain)		
Q 1 to 36	Interval scale (6-point Likert scale)	1=strongly disagree; 2=moderately disagree; 3=slightly disagree; 4=slightly agree; 5=moderately agree; 6=strongly agree For individual domains, scores of 4‐12 indicate dissatisfaction, 13‐16 reflect ambivalence, and 17‐24 represent satisfaction. Overall scores of 36‐108 indicate dissatisfaction, 109‐144 reflect ambivalence, and 145‐216 indicate satisfaction
Section D: Turnover Intention (TIS-6) (6 items)		
D1, 3, 4, 6	Interval scale (5-item Likert scale)	1=never to 5=always
D2	Interval scale	1=to no extent to 5=very large extent
D5	Interval scale	1=highly unlikely to 5=highly likely A total score below 18 reflects a desire to remain with the MOH^b^, whereas a score above 18 indicates a desire to leave.
Section E: baseline characteristics (12 items)		
E 1 to 12	Category scale	Age, gender, marital status, number of children, year of service in MOH, specialty and subspecialty, year of service as a specialist, grade, place of work, retirement scheme: Employees Provident Fund or pension, and the status of National Specialist Register

a WRSs: workforce retention strategies.

b MOH: Ministry of Health.

#### REDCap Survey Administration Workflow

REDCap will be implemented through a structured and systematic process that includes automated survey distribution, real-time response monitoring, built-in data validation rules, and secure data export procedures. These features aim to ensure accurate, consistent, and efficient data collection throughout all phases of the study. Table 3 shows each stage of the REDCap-based workflow, highlighting the specific tools used and their roles in the data collection process.

**Table 3.** Application of REDCap in a nationwide online cross-sectional study: key steps and implementation process.

Stage and steps	Description	Unique features	REDCap^a^ function used
Study design and questionnaire			
Project creation in REDCap	A REDCap project will be initiated to provide a secure and structured system for national-level survey deployment targeting MOH^b^ specialists.	Enables secure data storage and standardization across all survey responses.	Project setup and survey module activation
Questionnaire development	The instrument will be developed using the Online Designer, incorporating Likert-type and open-ended questions with embedded validation rules to prevent data entry errors.	Supports uniformity and improves data reliability.	Online Designer and Field Validation Rules
Branching logic and standardization	Branching logic will ensure that only relevant questions appear based on prior responses. For instance, respondents unaware of a particular WRS^c^ will skip related questions. Required fields will also be enforced to ensure data completeness.	Minimizes respondent burden and improves data quality.	Branching Logic and Required Fields
Management of study participants			
Importing selected sample	A systematically selected sample of MOH specialists will be imported to restrict access to eligible participants only.	Preserves data integrity and study eligibility criteria	Data import tool
Survey distribution and data collection			
Automated email invitations	Personalized email invitations with unique, nontransferable survey links are used for secure survey access and management of invitations and reminders. Survey responses cannot be linked to email addresses.	Ensures controlled access, supports reminder scheduling, and maintains participant anonymity	REDCap automated survey invitation module and participant list (anonymous mode)
Survey invitation log	This log will be used to monitor the distribution status of survey invitations and reminders to support targeted follow-up.	Mitigate potential nonresponse bias by enabling targeted follow-up	Survey invitation log
Follow-up reminders	Automated reminders will be sent during the second and third weeks to nonrespondents. Reminder emails will cease upon survey completion.	Improves participation while avoiding redundancy.	Automated reminder scheduler
Real-time response monitoring and quality control			
Live response tracking	A real-time dashboard will monitor response rates across subgroups, such as specialty or setting (hospital vs nonhospital).	Enables dynamic monitoring and targeted outreach	Record status dashboard
Data validation and quality control	The system will prompt participants to complete all mandatory questions within each section before submission (1 section per page), with selected fields configured using predefined validation criteria.	Minimize incomplete and invalid data entries	Required and validation fields
Duplicate response detection	Duplicate responses are prevented by using a closed REDCap survey with unique, nontransferable invitation links, each allowing only a single completed submission per recipient.	Prevents duplicate submissions	Survey distribution tool and participant list
Survey accessibility and usability			
Multidevice compatibility	The survey will be optimized for use on desktops, tablets, and mobile devices to support flexible participation across health care contexts.	Increases accessibility and response convenience	Built-in responsive design (automatically enabled for all surveys)
Data analysis and reporting			
Data export and automated reporting	Survey data will be exported in IBM SPSS Statistics compatible format. No identifiable variables will be included in the dataset used for analysis.	Facilitates secure and structured data analysis	Data export tool and automated report generation

a REDCap: Research Electronic Data Capture.

b MOH: Ministry of Health.

c WRS: workforce retention strategy.

### Pilot Study

A pilot study will be conducted involving 133 MOH specialists, representing approximately 10% of the 1325 participants calculated for the main study. This sample size is deemed appropriate to evaluate multiple components of survey implementation, including questionnaire clarity, internal consistency of multi-item domains, and the operational feasibility of data collection procedures [29]. While smaller pilot samples (eg, 30‐50 participants) may be sufficient for testing usability or basic functionality, a larger sample is warranted in this context to support broader assessments, including psychometric performance and system readiness [29].

The purpose of the pilot study is to assess the anticipated response rate, questionnaire usability, and internal consistency of multi-item domains, as well as to confirm the clarity and cultural appropriateness of survey items. The pilot study will be conducted via preproduction testing using in-built REDCap features to ensure the clarity of all questions and the proper functioning of relevant technical elements, such as branching logic, field validation, and device responsiveness (mobile/desktop). This will establish the usability of the questionnaire and subsequently minimize response bias and data loss. In addition, the operational functionality of the REDCap platform will be tested, including automated email distribution and real-time response capture, replicating the workflow planned for the full-scale national survey. Any necessary modifications will be incorporated prior to national rollout to enhance overall study quality.

Internal consistency metrics (eg, Cronbach α) will be calculated to examine the reliability of multi-item domains and their suitability to the Malaysian public health care and cultural context. An α coefficient equal to or greater than 0.7 will be considered acceptable [30]. Domains with suboptimal internal consistency will be reviewed and revised accordingly. All procedures used in the pilot will mirror those of the main study.

### Data Collection

The survey will comprise 7 sequential pages: a participant information sheet (PIS), an informed consent form, and 5 questionnaire sections, as detailed in Table 2. Data will be collected through a structured, self-administered online survey using the REDCap platform, as detailed in Table 3.

Eligible MOH specialists will be invited to participate via email. To minimize selection bias, no prenotifications or prior announcements will be issued. Each invitation will include a cover letter outlining the study objectives and procedures, along with a unique, nontransferable hyperlink to access the survey. This individualized link will restrict access to the intended recipient and prevent duplicate or unauthorized responses.

Upon accessing the survey, participants will first review the PIS and provide informed consent using a nonidentifiable “yes/no” response option. Only participants who provide consent can proceed to the survey items. To ensure data completeness, all items will be mandatory and the survey will be administered in sections (1 section per page). Participants must complete and submit each section before proceeding to the next. Responses may be reviewed and amended prior to section submission; however, once a section is submitted, responses within that section cannot be modified. Participants who are unable to complete a section in 1 sitting may use the “Save & Return Later” function to resume at their convenience.

REDCap paradata (eg, Survey Invitation Log and Record Status Dashboard) will be used to track recruitment and support nonresponse assessment. Survey invitations will be issued through REDCap-generated emails. These will be routed through MOH institutional mail servers and delivered using a secure email infrastructure that adheres to institutional data governance protocols. Due to daily email limits imposed by the MOH system, invitations will be distributed in 3 consecutive monthly batches. Follow-up reminder emails will be sent to nonrespondents during the second and third weeks of each distribution month to optimize response rates.

To maximize participation and minimize nonresponse, several strategies will be implemented, including individualized email invitations, clear and concise study information, appropriate timing of survey distribution, and a concise, mobile-friendly questionnaire design to reduce respondent burden. Response patterns will be monitored throughout the survey period to identify subgroups with lower participation rates and to implement targeted follow-up, including additional reminders via REDCap where appropriate.

### Data Management and Analysis

Only completed questionnaires will be included in the analysis. Incomplete surveys will be excluded from the analytic dataset and recorded as noncomplete submissions. The completion rate will be calculated using REDCap survey logs, where the number of completed surveys is divided by the number of surveys started. The partial response rate will be calculated as the number of noncomplete submissions divided by the number of surveys started. Response rate will be calculated as the number of submitted surveys divided by the number of unique invitations sent (calculated sample), based on REDCap invitation and survey status logs. Response and completion rates will be tabulated and reported upon completion of data collection in accordance with CHERRIES (Checklist 1).

Quantitative data will be exported from the REDCap platform to IBM SPSS Statistics Version 29 for analysis. Descriptive statistics will be used to examine the level of awareness, participation, and perceived effectiveness of WRSs, as well as job satisfaction and turnover intention. Binary logistic regression models will be utilized to examine the associations between WRSs and workforce outcomes, namely job satisfaction and turnover intention. Independent variables in this study would include WRS engagement (operationalized into 3 domains: awareness, participation, and perceived effectiveness). Multivariable logistic regression models will adjust for potential confounders, including age, gender, professional grade, disciplines (surgical vs nonsurgical), service duration, facility type, and geographic regions. No subgroup analyses will be conducted; all covariates will be included as adjustment variables in the regression models. Finite-population correction will not be applied, as the primary analyses focus on regression-based association estimates.

To minimize potential nonresponse bias, respondent characteristics will be descriptively compared with the sampling frame on key variables. No weighting or postsurvey statistical correction will be applied, as access to all the necessary variables in our entire study population (8953 specialists) is limited; hence, we would not be able to accurately calculate the necessary weights. Under these conditions, poststratification or raking weights may introduce additional bias and inflate variance without improving representativeness.

Qualitative data from 2 open-ended questions will be imported into NVivo version 12 (Lumivero) for organization and detailed analysis. NVivo will assist in systematically coding responses and grouping them into broader themes, facilitating the generation of meaningful insights. The analytical purpose of these questions is to identify context-specific strategies, implementation gaps, and participation barriers that may not be captured by quantitative questionnaires. Open-ended responses will be analyzed using deductive thematic analysis to complement the quantitative component of the study. The deductively derived qualitative themes will be cross-referenced with quantitative data to provide contextual and explanatory insights into the quantitative findings. The process will adhere to CHERRIES principles to ensure transparency and thorough examination.

### Ethical Considerations

#### Ethics Approval

This study is registered with the National Medical Research Registry and has received ethical approval from the Medical Research and Ethics Committee, MOH Malaysia (23-03199-3VQ). All study procedures will be conducted in accordance with the Malaysian Guidelines for Good Clinical Practice [31] and the principles of the Declaration of Helsinki.

#### Consent to Participate

Informed consent will be obtained electronically prior to the survey participation. Participants will first be presented with PIS outlining the study objectives, procedures, potential risks and benefits, data handling arrangements, and assurances regarding data confidentiality. Consent will be recorded using a nonidentifiable “yes/no” response option embedded within the survey, and only participants who select “yes” will be able to proceed to the questionnaire. Participation is entirely voluntary, and participants may self-withdraw from the study at any time before the full survey submission without any consequences on their employment status, performance evaluations, or access to workplace benefits. Once the questionnaire has been submitted, withdrawal of data will not be possible, as no personal identifiers or traceable information are collected or linked to survey responses, rendering individual responses unidentifiable. This limitation will be explicitly stated in the PIS to ensure fully informed consent. No compensation will be given to participants.

#### Anonymization and Data Privacy

Survey invitations will be distributed using email addresses uploaded to the REDCap participant list for recruitment purposes only. Email addresses will be managed within the participant list for invitation and reminder purposes and will not be stored with, nor linked to, survey response data in the dataset exported for analysis.

No personal identifiers will be collected in the questionnaire. The Participant Identifier function will be disabled and Survey Response Status will be automatically set to “Anonymous,” such that responses are not directly identifiable to users within the project interface. As survey responses are anonymous, we will not be able to entertain any request for individual data withdrawal after submission of the completed questionnaire.

#### Data Security, Access, and Repository

All data will be handled confidentially and stored on secure National Institutes of Health Data Repository System servers for a minimum period of 3 years following study completion. Data governance adheres to the MOH Standard Operating Procedure on Maintenance, Archival and Disposal of Study and Non-Study Files, as required under Medical Research and Ethics Committee approval [32]. Upon completion of the mandated retention period, and where the data are no longer required for regulatory, audit, or research purposes, study records will be securely disposed of in accordance with approved MOH data destruction procedures. Access to the dataset will be restricted to authorized members of the study team and used solely for the purposes of this study. As such, the data are not publicly available.

## Results

The results will present descriptive summaries of participants’ sociodemographic characteristics, employment profiles, and levels of awareness, participation, and perceived effectiveness of the 12 MOH-implemented WRSs. Findings from multivariable regression analyses will report associations between WRS engagement and workforce outcomes, including job satisfaction and turnover intention. Findings from the open-ended survey items will be reported narratively, highlighting common themes related to perceived facilitators, barriers, and recommendations for improving WRS implementation, to contextualize the quantitative results. A statistical significance will be set at *P*<.05. Results will be reported in accordance with the STROBE guidelines.

As of the submission of this protocol, participant recruitment and data collection have been completed. The study is currently in the data analysis phase. It is expected that the data analysis will be completed in March 2026 and that the results will be published in June 2026.

## Discussion

### Anticipated Findings and Policy Implications

This study protocol is important as it systematically outlines a national cross-sectional study to examine the implementation of the 12 WRSs across MOH health care facilities, with the aim of assessing associations, if any, between WRS engagement, job satisfaction, and turnover intention among MOH specialists. By capturing national-level data on awareness, participation, and perceived effectiveness, the study can provide a nuanced view of how WRSs are experienced across diverse clinical settings within the MOH system. This research represents a foundational step toward evidence-informed policymaking aimed at strengthening specialist retention and supporting the human resource sustainability of Malaysia’s public health care system.

To our knowledge, this is the first national cross-sectional study focused on specialist engagement with a wide range of MOH-implemented WRSs, encompassing both centrally administered initiatives and those requiring self-initiation or managerial discretion. The study will provide contextual factors, such as institutional readiness, administrative support, and resource availability, which are associated with variations in uptake and perceived effectiveness. By analyzing the variability of implementation across MOH health care settings, the study will offer key insights into how current strategies align with the needs and expectations of specialists and how these strategies can be further refined for equitable and effective applications.

This protocol sets the stage for generating practical evidence that can inform policy revisions and the development of more targeted, context-sensitive retention strategies. Understanding the lived experiences of specialists with respect to WRSs will help identify which elements foster engagement, enhance job satisfaction, and ultimately reduce turnover. The findings from this study may also be used to guide the development of strategic frameworks and best practice models that can be scaled or adapted across different facility types and regions within MOH Malaysia. Moreover, capturing specialists’ perspectives will enhance the real-world relevance and applicability of the study’s recommendations.

Empowering specialists with well-structured, responsive, and transparent retention strategies is essential for sustaining motivation and workforce stability [14,33]. Insights from this study will inform institutional policies and operational improvements that contribute to a more supportive working environment. By identifying which strategies resonate most with specialists and why, this study has the potential to promote both individual well-being and systemwide efficiency. The potential benefits extend beyond workforce outcomes, including improvements in service continuity, patient care quality, and long-term cost savings through reduced recruitment and training burdens.

Finally, this study will highlight critical gaps in the implementation of WRSs, guiding future research and policy dialogue. It may also generate insights applicable to other health professional groups facing similar retention challenges. By advancing the evidence base for workforce retention in Malaysia’s public health system, this study offers a strategic opportunity to enhance human resource planning, specialist well-being, and health system resilience in the years to come.

### Integration of REDCap for National-Level Data Collection

This protocol strategically integrates REDCap to facilitate standardized, large-scale data collection from specialists across MOH Malaysia’s public health care system. Traditional survey methods, such as paper-based questionnaires or in-person interviews, often pose significant logistical and operational challenges, particularly in geographically dispersed or high-volume clinical settings [21]. These challenges include manual data entry errors, time delays, data loss risks, and the resource-intensive nature of deploying field staff [21]. In contrast, REDCap offers a secure, browser-based solution that enables centralized administration, efficient data capture, and real-time monitoring while maintaining strict data privacy and security standards [34].

The use of REDCap in this study enhances methodological rigor by enabling structured data entry through built-in features, such as branching logic, validation rules, and audit trails [35,36]. These features reduce data entry errors, ensure internal consistency, and support traceability throughout the research process. Compared to general-purpose platforms, such as Google Forms or SurveyMonkey, REDCap offers distinct advantages in data management, including compliance safeguards, user access controls, and robust metadata tracking [37]. These capabilities are especially valuable in health research, where maintaining data integrity and participant confidentiality is essential.

Moreover, REDCap is designed for interoperability with statistical and qualitative analysis software such as SPSS, STATA (StataCorp LLC), and NVivo, allowing for seamless data export and minimizing the need for manual data handling [20]. This streamlines the data cleaning and analysis workflow and ensures the production of high-quality datasets suitable for both quantitative and qualitative analyses. Its widespread adoption in international health research, including in low-resource and multisite studies, underscores REDCap’s scalability and adaptability in diverse research settings [21].

Incorporating REDCap also supports uniform implementation across all participating sites, fostering consistency and transparency in data collection procedures [21]. This is particularly crucial for a national-level study involving multiple facilities with varied institutional capacities. REDCap’s deployment is expected to enhance study replicability, improve coordination across research teams, and promote data governance standards aligned with international best practices. Prior evidence has shown that REDCap is well-received by clinicians and researchers alike, with studies reporting high levels of satisfaction related to its reliability, user-friendliness, and capacity to produce complete, accurate datasets [34]. As such, the integration of REDCap is a key enabler of this study’s operational feasibility and scientific integrity.

Beyond technical capabilities, ensuring transparency and ethical accountability in digital survey implementation is also a critical element of methodological integrity. This protocol emphasizes the study’s adherence to CHERRIES, a standardized reporting framework designed to enhance the transparency, reproducibility, and ethical standards of online research. CHERRIES’ compliance ensures the clear reporting of key survey elements, such as consent procedures, response rates, and data handling protocols, while facilitating cross-study comparisons and future meta-analyses [38]. In the context of this nationwide web-based survey, CHERRIES’ alignment reinforces the credibility of study findings and contributes to the broader goal of generating ethically sound and scientifically rigorous evidence within the evolving digital health research ecosystem.

### Application of Validated Instruments to Support Methodological Rigor in Online Research

To ensure robust and credible measurement of workforce retention dynamics, this study employs a validated, multidimensional set of instruments to examine associations between MOH-implemented WRS, job satisfaction, and turnover intention among specialists in MOH Malaysia’s public health care system. Specifically, the study integrates 3 well-established instruments: adapted items from Belbin et al [15] to assess WRS perceptions, the TIS-6, and the JSS. The combined use of these instruments provides a methodologically sound framework to examine relationships between organizational strategies and individual-level outcomes, consistent with best practices in health workforce research [15,26,28].

The TIS-6 is a brief, psychometrically validated scale widely used in health workforce research to assess an individual’s intention to leave their current role. It captures both affective and cognitive components of turnover intention, offering insight into short- and longer-term attrition risks [39]. Its brevity and clarity make it particularly suitable for clinician populations, who often face time constraints in research participation. Previous validation studies have confirmed the tool’s internal consistency (Cronbach α=0.80) and predictive validity, further supporting its applicability in large-scale health care settings [28].

To measure job satisfaction, this study adopts the JSS, a comprehensive instrument developed to assess satisfaction across 9 key domains of 4 items each, including pay, promotion, supervision, fringe benefits, contingent rewards, operating procedures, coworkers, nature of work, and communication [26,27]. The JSS has demonstrated robust psychometric properties in diverse health systems and cultural settings, including countries such as Malaysia, Jordan, Singapore, Iran, Turkey, and the United States [13,40]. Its multidimensional structure allows for a granular understanding of the factors contributing to professional fulfillment or dissatisfaction among specialists. A systematic review conducted in 2003 reaffirmed its internal consistency and construct validity, underscoring its continued relevance for cross-cultural health care research [41].

### Limitations

Several limitations must be acknowledged. First, the reliance on self-reported data may introduce response or recall bias, although anonymity, neutral phrasing, and piloting are expected to mitigate these effects. Second, nonresponse bias remains a concern, particularly among specialists facing high clinical demands. Furthermore, it may be further complicated by the use of mandatory survey items and the exclusion of partially completed questionnaires. To mitigate this risk, practical steps were incorporated to encourage participation, and potential nonresponse bias will be assessed through the descriptive comparison of respondent characteristics with those available from the sampling frame on key variables. Finally, although open-ended items allow for limited qualitative exploration, they may not capture the same depth achievable through interviews or focus group discussions.

### Future Directions

This study protocol establishes a foundation for ongoing, data-driven monitoring of WRSs within the MOH health care system. Future research may adopt a mixed methods design incorporating more intensive qualitative methods to deepen insights into WRS experiences. Longitudinal designs are also warranted to assess the long-term impact of evolving WRS policies on specialist retention. Comparative studies between public and private sector settings may also yield valuable insights into contextual differences in WRS effectiveness. In addition, integrating REDCap dashboards into MOH human resource planning systems could support real-time surveillance and predictive modeling of retention risks, enabling more targeted and evidence-informed decision-making.

### Conclusion

This study protocol presents a comprehensive framework for the national-level cross-sectional study of WRS targeting specialists within the MOH, Malaysia. By examining the association between WRS implementation, job satisfaction, and turnover intentions, the study seeks to align WRSs more closely with the realities of clinical practice and workforce expectations. The integration of REDCap ensures efficient, secure, and standardized data collection, while adherence to CHERRIES guidelines strengthens methodological transparency and ethical rigor. By uncovering context-specific barriers and enablers, the study offers practical insights into the implementation and effectiveness of existing WRSs, providing a foundation for more responsive and targeted policy interventions to address specialist attrition. In doing so, it supports evidence-informed decision-making among stakeholders in MOH Malaysia and contributes to broader efforts to mitigate brain drain. Ultimately, this protocol advances the long-term goal of building a resilient, motivated, and sustainable health workforce, essential for the continued strengthening of Malaysia’s public health care system.

## Supplementary material

10.2196/83377 Multimedia Appendix 1 List of workforce retention strategies for physicians and dentists in the Ministry of Health Malaysia.

10.2196/83377 Checklist 1 CHERRIES checklist.

## Abbreviations

CHERRIES: Checklist for Reporting Results of Internet E-Surveys
HRMIS: Human Resource Management Information System
JSS: Job Satisfaction Survey
MOH: Ministry of Health
PIS: participant information sheet
REDCap: Research Electronic Data Capture
STROBE: Strengthening the Reporting of Observational Studies in Epidemiology
TIS-6: Turnover Intention Scale-6
WRS: workforce retention strategy
