# Supplementary material for: Specialists’ Perceptions of Workforce Retention Strategies in the Malaysian Ministry of Health and Their Association With Job Satisfaction and Turnover Intention: Protocol for a REDCap-Based National Cross-Sectional Survey
Source: JMIR Res Protoc. 2026 Apr 1;15:e83377. doi: 10.2196/83377 (PMC13043007; doi:10.2196/83377)
Supplement: Multimedia Appendix 1 [file resprot-v15-e83377-s001.docx]

**Appendix 1**

**List of Workforce Retention Strategies (WRS) for Physicians and Dentists in the MOH Malaysia**

| **No** | **Incentives** | **Description** |
| --- | --- | --- |
| 1. | Specialist Incentive Allowance | ● Paid to Medical and Dental Specialist (clinical/public health) to appreciate the contributions and skills of specialists. |
| 2. | Elective Surgery Allowance | ● Paid to Specialists & Medical / Dental Officers who perform elective surgery on Saturdays from 8.00 AM to 1.00 PM at government hospitals.  ● The maximum rate paid is for 5 hours and does not depend on the time the surgery is completed. |
| 3. | On-Call Duty Allowance for Medical Officers  (Outside Regular Working Hours Allowance) | ● Paid to Medical / Dental Officers who are instructed to perform on-call duties (on-call/night call) and work continuously in hospital wards and clinics after working hours usually to ensure health and medical services to patients are not affected.  ● Allowance rates differ between specialists and non-specialists, type of on-call duty (passive / active), type of day (weekday / public holiday / weekend) and duration of duty (more than 15 hours / more than 4 hours / less than 4 hours). |
| 4. | Placement Incentive Payment for Medical and Dental Specialist in Health Facilities in Sabah, Sarawak & Labuan  (Specialist Placement Incentive Payment) | ● Paid to Medical and Dental Specialist (Clinical / Public Health) who work in health facilities (State Health Department, Health Office, Dental Office, hospitals, health clinics, dental clinics, rural clinics, community clinics, mobile clinics) in Sabah, Sarawak, and Labuan.    ● Paid proportionately in circumstances where the service period is not an even one (1) calendar month. |
| 5. | Service Fees to MOH Specialists and Medical Officers Who Treated Patients at National Heart Institute Sdn. Bhd. (IJNSB) | ● Physicians and Medical Officers from related disciplines are allowed to provide treatment at IJNSB.    ● MOH Physicians treating cases referred by IJNSB are paid for their services, subject to stipulated conditions.    ● Services provided are examination and treatment consultation, certain procedures, and daily follow-up examinations. |
| 6. | Time-based Promotion & Creation of Grade 56 for Specialist | ● Career paths of Medical Officers and Dental Officers were improved with time-based promotion.    ● Promotion period from Grade 41 to 54 for the specialist route is only 9 years compared to 12 years for the non-specialist route, 14 years for Pharmacy Officers and 25 years for Education Service Officers (Teachers). Promotion for other service schemes is not time-based and according to vacancies.    ● Grade 56 was created specifically for Physicians and Dentists with an allowance rate that is almost the same as the Top Management group (JUSA). |
| 7. | Flexible Working Hours  (1 day a week) | ● Physicians Grade UD54 and above at MOH are given flexibility of one day a week (during official working hours) to exercise Flexible Working Hours to carry out activities within MOH facilities (medical research activities and teaching) as well working in the private sector.    ● This flexibility is to bridge the income gap between Government and Private Physicians, increasing the number of researchers in the field of medicine and health, as well as encouraging and stimulating knowledge transfer to medical and health trainees. |
| 8. | Full Paying Patient Scheme (FPP) | ● An initiative to increase the income source of Physicians in hospitals to reduce the income gap between Government and Private Physicians. This indirectly reduces the migration of MOH Physicians to private hospitals.    ● Part of the income earned by the hospital through the FPP scheme will be given to Physicians registered under this service. Thus, competitive rewards can be offered to Physicians to continue serving in Government hospitals.    ● Distribution of payments received from FPP patients to Physicians is between 50% to 100% for different types of fees such as consultation, investigation, procedure, treatment, medical report and medical examination package. |
| 9. | Locum at a private health facility | ● Medical Officers and Dental Officers are allowed to do outside work (locum) and can be carried out after office hours, on public holidays, weekends, annual leave or during study leave. |
| 10. | Locum at MOH health facilities | ● Medical Officers can run locums at MOH facilities through the implementation of an after-hours patient treatment clinic in the Emergency Department.    ● Pilot projects, as initiatives to reduce overcrowding in Government hospitals, are done by extending operating hours of six (6) Health Clinics, over a period of six (6) months. Medical Officers involved in these projects on Weekly Holidays (Sunday/Friday) are also given Locum Allowance. |
| 11. | Short-term training opportunities abroad | ● MOH staff including Physicians and Dentists have the opportunity to participate in short-term training service abroad, with expenses covered including meal and daily allowances, travel fare and air ticket cost, hotel fee, warm clothing allowance, medical expenses and various expenses. |
| 12. | Opportunity to continue sub-specialty studies | ● Physicians with recognised specialty qualifications and experience of at least 24 months after being gazetted, can apply for sub-specialty studies with study leave and scholarships from the Government.    ● Sub-specialty programmes can be undertaken through three methods:   - - 1. combination of domestic and foreign studies: Period of 2-3 years studying in the country with Consultant’s supervision and additional exposure abroad for 1 year;     2. studying entirely in the country: Period of 3-4 years at MOH’s hospital, university hospital or identified training centre; or     3. studying completely abroad (area of interest): Period of 12 months in an overseas institution in a related field |

Source: Human Resource Department, MOH Malaysia 2022
