# Supplementary material for: Specialists’ Perceptions of Workforce Retention Strategies in the Malaysian Ministry of Health and Their Association With Job Satisfaction and Turnover Intention: Protocol for a REDCap-Based National Cross-Sectional Survey
Source: JMIR Res Protoc. 2026 Apr 1;15:e83377. doi: 10.2196/83377 (PMC13043007; doi:10.2196/83377)
Supplement: Checklist 1 [file resprot-v15-e83377-s002.docx]

**Checklist for Reporting Results of Internet E-Surveys (CHERRIES)**

| **Item Category** | **Checklist item** | **Explanation** | **Response** |
| --- | --- | --- | --- |
| **Design** | Describe survey design | Describe the target population and sample frame. Is the sample a convenience sample? (In “open” surveys this is most likely.) | The study population will include all specialists within the Ministry of Health (MOH), identified through the Human Resource Management Information System 2.0 (HRMIS 2.0) until December 2023. The sample frame will consist of 1,325 active specialists which will be selected through systematic random sampling from a list of 8,953 active MOH specialists. |
| **IRB (Institutional Review Board) approval and informed consent process** | IRB approval | Mention whether the study has been approved by an IRB. | This study is registered with the National Medical Research Registry and has received ethical approval from the Medical Research and Ethics Committee (MREC), MOH Malaysia (NMRR-23-03199-3VQ [IIR]). |
|  | Informed consent | Describe the informed consent process. Where were the participants told the length of time of the survey, which data were stored and where and for how long, who the investigator was, and the purpose of the study? | Participants will review the PIS on the first survey page and provide informed consent via a non-identifiable “Yes/No” response option before accessing the questionnaire. Survey duration, study purpose, investigator details, data collected, data storage location and retention period, confidentiality safeguards, and withdrawal conditions will be described in the PIS. |
|  | Data protection | If any personal information was collected or stored, describe what mechanisms were used to protect unauthorized access. | Email addresses will be managed within the Participant List for invitation and reminder purposes and will not be stored with, nor linked to, survey response data in the dataset exported for analysis. No personal identifiers will be collected in the questionnaire, the Participant Identifier function will be disabled, and Survey Response Status will be automatically set to “Anonymous,” such that responses are not directly identifiable to users within the project interface. |
| **Development and pre-testing** | Development and testing | State how the survey was developed, including whether the usability and technical functionality of the electronic questionnaire had been tested before fielding the questionnaire. | The WRS questionnaire will adapt items from Belbin et al. (2012) and contextualise them for the local healthcare environment, aligning with the study objectives. Additionally, validated tools like Spector’s Job Satisfaction Survey (JSS) and the Turnover Intention Scale (TIS-6) will be incorporated for robust measurement. Usability and technical functionality will be tested prior to fielding through pilot pre-production testing using in-built REDCap features, with refinements will be made before national rollout. |
| **Recruitment process and description of the sample having access to the questionnaire** | Open survey versus closed survey | An “open survey” is a survey open for each visitor of a site, while a closed survey is only open to a sample that the investigator knows (password-protected survey). | The survey will be a closed online survey, accessible only to the identified sample through personalised email invitations containing unique access links. The survey link will not be publicly accessible, ensuring controlled participation. |
|  | Contact mode | Indicate whether or not the initial contact with the potential participants was made on the Internet. (Investigators may also send out questionnaires by mail and allow for Web-based data entry.) | Initial contact with potential participants will be made via email using institutional email addresses uploaded to the REDCap Participant List. No external advertisements or public announcements. |
|  | Advertising the survey | How/where was the survey announced or advertised? Some examples are offline media (newspapers), or online (mailing lists – If yes, which ones?) or banner ads (Where were these banner ads posted and what did they look like?). It is important to know the wording of the announcement as it will heavily influence who chooses to participate. Ideally, the survey announcement should be published as an appendix. | The survey will not be publicly announced or advertised. Participation will be by direct invitation only, with survey links sent via official Ministry of Health (MOH) email communications to selected specialists identified through the Human Resource Management Information System (HRMIS 2.0). No offline media, public advertisements, banner ads, or external online platforms will be used. |
| **Survey administration** | Web/E-mail | State the type of e-survey (eg, one posted on a Web site, or one sent out through e-mail). If it is an e-mail survey, were the responses entered manually into a database, or was there an automatic method for capturing responses? | The survey will be conducted as a closed survey, distributed via personalised email invitations to selected participants using REDCap platform. Responses will be captured automatically through the REDCap platform, eliminating the need for manual data entry and ensuring data accuracy and security. |
|  | Context | Describe the Web site (for mailing list/newsgroup) on which the survey was posted. What is the Web site about, who is visiting it, what are visitors normally looking for? Discuss to what degree the content of the Web site could pre-select the sample or influence the results. For example, a survey about vaccination on an anti-immunization Web site will have different results from a Web survey conducted on a government Web site | Not applicable. The survey will not be posted on any website, mailing list, or newsgroup. Instead, it will be distributed exclusively via the official Ministry of Health (MOH) email system to selected participants that will be identified through HRMIS 2.0. |
|  | Mandatory/ voluntary | Was it a mandatory survey to be filled in by every visitor who wanted to enter the Web site, or was it a voluntary survey? | Not applicable to website access. Participation in the survey will be entirely voluntary. The survey will be distributed via invitation-only official MOH email, and participation will not be mandatory. Participants may decline to participate or withdraw at any point prior to submission without any consequences. |
|  | Incentives | Were any incentives offered (eg, monetary, prizes, or non-monetary incentives such as an offer to provide the survey results)? | No monetary or non-monetary incentives will be offered. |
|  | Time/Date | In what timeframe were the data collected? | The survey will be conducted over three months, with periodic reminders sent to maximise participation. |
|  | Randomisation of items or questionnaires | To prevent biases items can be randomised or alternated. | The questionnaire will be structured to address the study's key objectives. It includes both quantitative and open-ended questions to allow comprehensive analysis. Questions will be presented sequentially to guide respondents through the survey logically. Randomisation is not required as it will not provide any additional benefits to the survey. |
|  | Adaptive questioning | Use adaptive questioning (certain items, or only conditionally displayed based on responses to other items) to reduce the number and complexity of the questions. | Adaptive questioning will be used, where certain items will be conditionally displayed based on prior responses to minimise questionnaire length and enhance the response experience. |
|  | Number of Items | What was the number of questionnaire items per page? The number of items is an important factor in the completion rate. | The survey will consist of a manageable number of items per page to minimise respondent fatigue. |
|  | Number of screens (pages) | Over how many pages was the questionnaire distributed? The number of items is an important factor in the completion rate. | The questionnaire will be distributed across seven pages, with each page containing a balanced number of items to facilitate respondent engagement and minimise survey fatigue. |
|  | Completeness check | It is technically possible to do consistency or completeness checks before the questionnaire is submitted. Was this done, and if “yes”, how (usually JAVAScript)? An alternative is to check for completeness after the questionnaire has been submitted (and highlight mandatory items). If this has been done, it should be reported. All items should provide a non-response option such as “not applicable” or “rather not say”, and the selection of one response option should be enforced. | Automated completeness checks will be implemented within REDCap to ensure mandatory fields are completed before submission. |
|  | Review step | State whether respondents were able to review and change their answers (eg, through a Back button or a Review step which displays a summary of the responses and asks the respondents if they are correct). | Respondents will have the ability to review and edit their responses before submission. |
| **Response rates** | Unique site visitor | If you provide view rates or participation rates, you need to define how you determine a unique visitor. There are different techniques available, based on IP addresses or cookies, or both. | No tracking via IP addresses or cookies will be utilised in REDCap, in compliance with data privacy policies and to maintain respondent confidentiality. |
|  | View rate (Ratio of unique survey visitors/unique site visitors) | Requires counting unique visitors to the first page of the survey, divided by the number of unique site visitors (not page views!). It is not unusual to have view rates of less than 0.1 % if the survey is voluntary. | We are unable to determine how many individuals viewed the online survey, as unique survey page views are not available as a reportable metric in our institutional REDCap configuration. The system records an entry only when a participant submits the first page of the survey. In this study, the first page will be the Participant Information Sheet (PIS). As such, the earliest measurable point of survey access will be the number of individuals who submitted the PIS. Similar limitations in reporting view rates have been described in prior REDCap-based surveys [42] |
|  | Participation rate (Ratio of unique visitors who agreed to participate/unique first survey page visitors) | Count the unique number of people who filled in the first survey page (or agreed to participate, for example by checking a checkbox), divided by visitors who visit the first page of the survey (or the informed consent page, if present). This can also be called the “recruitment” rate. | The participation rate will be reported as the number of participants who provided consent divided by the number of participants who submitted the first page (PIS), which represents the earliest measurable point of survey access in our REDCap configuration. |
|  | Completion rate (Ratio of users who finished the survey/users who agreed to participate) | The number of people submitting the last questionnaire page is divided by the number of people who agreed to participate (or submitted the first survey page). This is only relevant if there is a separate “informed consent” page or if the survey goes over several pages. This is a measure of attrition. Note that “completion” can involve leaving questionnaire items blank. This is not a measure of how completely questionnaires were filled in. (If you need a measure for this, use the word “completeness rate”.) | Completion rate will be calculated using REDCap survey logs as the number of completed surveys divided by the number of surveys started. |
| **Preventing multiple entries from the same individual** | Cookies used | Indicate whether cookies were used to assign a unique user identifier to each client computer. If so, mention the page on which the cookie was set and read, and how long the cookie was valid. Were duplicate entries avoided by preventing users access to the survey twice; or were duplicate database entries having the same user ID eliminated before analysis? In the latter case, which entries were kept for analysis (eg, the first entry or the most recent)? | Cookies will not be used to identify duplicate entries. |
|  | IP check | Indicate whether the IP address of the client computer was used to identify potential duplicate entries from the same user. If so, mention the period for which no two entries from the same IP address were allowed (eg, 24 hours). Were duplicate entries avoided by preventing users with the same IP address access to the survey twice; or were duplicate database entries having the same IP address within a given period eliminated before analysis? If the latter, which entries were kept for analysis (eg, the first entry or the most recent)? | IP addresses will not be used to identify duplicate entries. |
|  | Log file analysis | Indicate whether other techniques to analyze the log file for identification of multiple entries were used. If so, please describe. | Log file analysis will not be used to identify duplicate entries. |
|  | Registration | In “closed” (non-open) surveys, users need to log in first and it is easier to prevent duplicate entries from the same user. Describe how this was done. For example, was the survey never displayed a second time once the user had filled it in, or was the username stored together with the survey results and later eliminated? If the latter, which entries were kept for analysis (eg, the first entry or the most recent)? | Each participant will receive a unique survey link via email to track individual responses. REDCap will prevent multiple submissions by restricting survey access once a response has been submitted. |
| **Analysis** | Handling of incomplete questionnaires | Were only completed questionnaires analyzed? Were questionnaires that terminated early (where, for example, users did not go through all questionnaire pages) also analyzed? | The final analysis will include only fully completed questionnaires. Partial responses will be excluded, and data completeness will be assessed based on submission status. |
|  | Questionnaires submitted with an atypical timestamp | Some investigators may measure the time people needed to fill in a questionnaire and exclude questionnaires that were submitted too soon. Specify the timeframe that was used as a cut-off point, and describe how this point was determined. | We will use REDCap timestamps to estimate survey completion time. Based on the median completion time from the pilot study, a lower cut-off point will be applied, and responses completed faster than this will be excluded as likely “speeding” to ensure data quality. |
|  | Statistical correction | Indicate whether any methods such as weighting of items or propensity scores have been used to adjust for the non-representative sample; if so, please describe the methods. | No weighting or statistical correction for non-representative samples will be applied. Although systematic random sampling ensures equal probability of selection at the invitation stage, the study team does not have access to complete auxiliary variables for the entire specialist population (N = 8,953) that would be required to construct reliable non-response weights. Applying post-stratification or raking under these conditions would introduce additional bias and inflate variance without improving representativeness. |
